# Small target detection with remote sensing images based on an improved YOLOv5 algorithm

**DOI:** 10.3389/fnbot.2022.1074862

**Published:** 2023-02-08

**Authors:** Wenjing Pei, Zhanhao Shi, Kai Gong

**Affiliations:** ^1^The Seventh Research Division and the Center for Information and Control, School of Automation Science and Electrical Engineering, Beihang University (BUAA), Beijing, China; ^2^School of Information Science and Engineering, Shandong Agriculture and Engineering University, Jinan, China

**Keywords:** small target detection, remote sensing images, YOLOv5s, deep learning, EIoU loss

## Abstract

**Introduction:**

Small target detection with remote sensing images is a challenging topic due to the small size of the targets, complex, and fuzzy backgrounds.

**Methods:**

In this study, a new detection algorithm is proposed based on the YOLOv5s algorithm for small target detection. The data enhancement strategy based on the mosaic operation is applied to expand the remote image training sets so as to diversify the datasets. First, the lightweight and stable feature extraction module (LSM) and C3 modules are combined to form the feature extraction module, called as LCB module, to extract more features in the remote sensing images. Multi-scale feature fusion is realized based on the Res 2 unit, Dres 2, and Spatial Pyramid Pooling Small (SPPS) models, so that the receptive field can be increased to obtain more multi-scale global information based on Dres2 and retain the obtained feature information of the small targets accordingly. Furthermore, the input size and output size of the network are increased and set in different scales considering the relatively less target features in the remote images. Besides, the Efficient Intersection over Union (EIoU) loss is used as the loss function to increase the training convergence velocity of the model and improve the accurate regression of the model.

**Results and discussion:**

The DIOR-VAS and Visdrone2019 datasets are selected in the experiments, while the ablation and comparison experiments are performed with five popular target detection algorithms to verify the effectiveness of the proposed small target detection method.

## 1. Introduction

With the development of remote sensing technologies, a large amount of remote sensing images can be obtained from video satellites and unmanned aerial vehicles (UAVs) (Hu et al., 2019; Zhang et al., 2019; Hou et al., 2020; Lu et al., 2020; Wang et al., 2020; Pei and Lu, 2022). Recently, remote sensing image processing has attracted widespread attention, such as target detection, classification, tracking, and surveillance (Jia, 2000, 2003; Guo et al., 2017; Wang et al., 2018; Yin et al., 2020; Zhong et al., 2020; Jiang et al., 2021; Dong et al., 2022; Habibzadeh et al., 2022; Ma and Wang, 2022; Pei, 2022). Particularly, target detection is a hot topic with remote sensing images (TDRSIs), where the TDRSI has been widely applied in the fields of military, transportation, forest survey, security monitoring, disaster monitoring, and so on (Zhang et al., 2016; Han et al., 2017; Zhu et al., 2017). Therefore, TDRSI is a significant and challenging task due to the small size of the targets, high speed detection, and high accuracy requirements (Zhang et al., 2017; Dong et al., 2022).

Target detection aims to find all interested objects in the images, which has been studied with the development of computer vision technologies in recent decades. Numerous algorithms, especially convolutional neural networks (CNNs), have been widely employed for general target detection, such as SSD, YOLO, R-CNN, and Faster R-CNN (He et al., 2016; Li et al., 2019, 2021; Zhong et al., 2020; Fan et al., 2021; Tu et al., 2021; Dong et al., 2022; Mikriukov et al., 2022). For instance, Lawal (2021) have proposed a modified YOLOv3 model to detect tomatoes in complex environments. Wu et al. (2018) presented a different scaled algorithm based on the Faster R-CNN to solve small-scaled face detection. YOLOv3 network can be used for blood cell recognition (Shakarami et al., 2021) while a YOLOv4 algorithm can be used for oil well detection (Shi et al., 2021).

**Figure 1:**
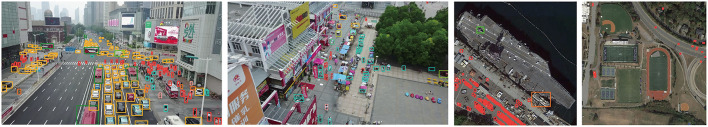
Examples of targets in remote sensing images.

Considering general target detection, small target detection in remote sensing images is more difficult due to several reasons (refer to Figure 1) (Meng, 2012; Li, 2016; Du et al., 2018; Chen et al., 2021; Liu et al., 2022). First, the scales of the remote sensing images may be relatively large compared to the small target size or clustered targets in the images. Moreover, the background of the remote sensing images could be complex and fuzzy, sometimes even similar to the target features. Third, there is not enough feature information of the targets in one image, i.e., vehicles, pedestrians, and others have only few pixels for object detection in the optical remote sensing images (DIOR) (Li et al., 2020) and Visdrone2019 (Zhu et al., 2019) datasets.

Hence, a lot of methods have been developed specifically to achieve small target detection in remote sensing images. For instance, Lu et al. (2021) have proposed a single shot detection (SSD) to detect the small target with complex background and scale variations. An improved YOLOv3 model has been designed for ship detection in remote sensing images with high accuracy and robustness (Xu, 2020). In Wang J. et al. (2020), an end-to-end feature-reflowing pyramid network has been proposed for multi-scale and multi-class object detection. Furthermore, a novel cascaded rotating-anchor-assisted detection network has been presented in Yu et al. (2022) to improve ship detection accuracy with aerial images. Moreover, Huang et al. designed a lightweight target detector to rapidly and accurately detect small targets (Huang et al., 2022). A detection algorithm based on the feature balancing and refinement network is developed to successfully detect ships (Fu et al., 2020). A squeeze-and-excitation YOLOv3 algorithm has been designed for small target detection in remote sensing images with low computation costs (Zhou et al., 2021). Moreover, Ling et al. (2022) have developed a new time-delay feedback model to detect small target motion in complex dynamic backgrounds. An indoor small target detection algorithm is described in Huang L. et al. (2022) based on multi-scale feature fusion to improve the accuracy and speed of the target detection.

Based on the above analysis, this study presents an improved LCB-YOLOv5s detection algorithm for remote sensing images. First, a new module comprising the lightweight and stable module (LSM) and cross-stage partial networks with three convolutions (C3) structure module where these modules are combined to form the feature extraction module, called as LCB module, is designed to extract numerous features of small targets. Then, the Spatial Pyramid Pooling Small (SPPS) module is developed to increase the weight of these features in the spatial dimension. Moreover, the Duble Res2Net (Dres2) module is used in the head to increase the receptive field so as to obtain more multi-scale global information and realize fine-grained feature extraction. In order to overcome the difficulty of relatively few features, the input size of the network is increased with different output feature map sizes. In summary, the contributions of the paper are summarized as follows:

1. An LCB-YOLOv5 algorithm has been developed for small target detection with remote sensing images. In the feature extraction module, the LCB module is configured based on the LSM and C3 modules to extract more features. Moreover, the SPPS and Dres2 modules are introduced to increase the weight of the features in the receptive field and so as to extract more multi-scale global information.
2. In order to improve the accuracy of the small target detection, the input size of the network is increased from 640 × 640 to 1,024 × 1,024, and the output feature map size is set as 32 × 32, 64 × 64, and 128 × 128, respectively.
3. The *EIoU* function is employed as the loss function to increase the training convergence velocity of the model and the regression accuracy for the target detection.

The remainder of the paper is organized as follows. Section 2 describes the proposed method in detail. Experiments of the small target detection with the selected datasets are performed and the results are analyzed in Section 3. The conclusion is provided in Section 4.

## 2. The proposed method

**Figure 2:**
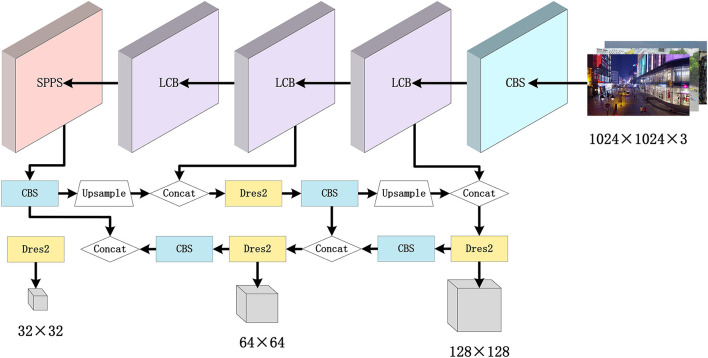
The architecture of the developed LCB-YOLOv5s.

This section presents the details of the proposed method. As shown in Figure 2, except for the first layer, the 3 × 3 convolutional layers in the backbone of the YOLOv5s detection algorithm are replaced with the LSM module. Since small targets have fewer features than those large targets in the images, the SPPS module is designed to increase the weight of these features in the spatial dimension. The Dres2 module is further introduced in the head with the strategy of multi-scale feature fusion to enhance the small target detection performance. The input size of the network is also increased with various output feature map sizes, while the EIoU loss function is designed to increase the convergence speed.

### 2.1. Data augmentation

**Figure 3:**
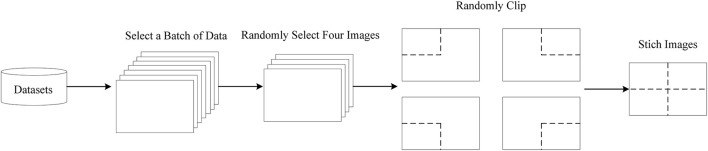
The procedure of the mosaic data enhancement.

In general, the original training data have to be pre-processed to meet the training requirement; hence, many data enhancement strategies are employed to expand and diversify the remote sensing images so as to improve the generalization ability of the trained model and to minimize the irrelevant characteristic information in the training data. As shown in Figure 3, the mosaic operation is applied to enrich the datasets, where four original images can be randomly selected from a batch in the datasets to perform a flip, translate, change the color gamut, and stitch the images such operations. Based on the above data enhancement operations, the size of the images is relatively close to the small targets, and the number of small targets can be increased in the remote sensing images. Therefore, the small target datasets can be expanded, which can effectively improve the small target detection ability of the model. Accordingly, the demand for GPU memory can be reduced and the training speed can be also improved greatly.

### 2.2. Feature extraction module

**Figure 4:**
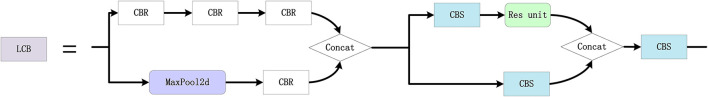
The structure of the LCB module.

In the remote sensing images, the sizes of the targets may be small and the edges of the targets may be blurred. Hence, a LCB feature extraction module is designed to improve the target detection performance, as shown in Figure 4. Specifically, numerous features of the small targets can be extracted using the LSM module. The standard 3 × 3 convolution is used for feature extraction, and some significant features of the original feature map are preserved using maximum pooling. Then, the output feature map is enriched by concatenation. Moreover, the C3 module can perform feature extraction and fusion, where 1 × 1 convolution is applied to reduce the dimension of the original feature map, and the feature map after convolutional extraction is spliced as the output.

**Figure 5:**
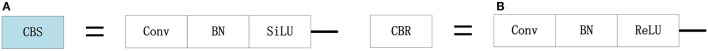
The schematic diagram of the standard convolution: **(A)** CBS module, **(B)** CBR module.

It is known that the *conv* + *batch normalization* + *silu* (CBS) and *conv* + *batch normalization* + *relu* (CBR) modules are two types of standard convolution modules. As shown in Figures 5A, B, CBS and CBR utilize the convolution operation, batch normalization (BN), and activation function, where the SiLU and the ReLU are employed as the activation functions, respectively. It is noted that the CBR module with the ReLU can reduce the amount of calculation and eliminate the gradient diminishing, where the activating function with ReLu can learn faster than the sigmoid or tanh functions.

**Figure 6:**
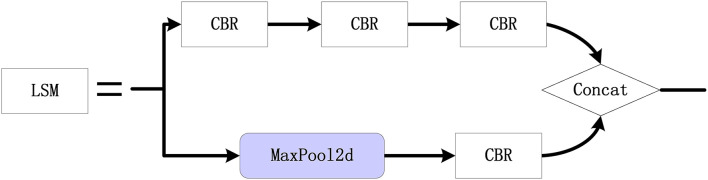
LSM module configuration.

Figure 6 displays the proposed LSM module, mainly composed of convolution and pooling branches. First, the 1 × 1 standard convolution and 3 × 3 convolution are used to reduce the data dimension and extract features, respectively. Then, the 1 × 1 standard convolution is used once again to increase the data dimension. Furthermore, the feature map is subsampled by 2 × 2 max pooling and the number of channels is adjusted based on the 1 × 1 standard convolution. Finally, the output is obtained based on the Concat module with the above features. Compared to the traditional 3 × 3 convolution, LSM can obtain more abundant features. On the other hand, LSM can preserve some significant features of the original feature map based on the maximum pooling. On the other hand, LSM can enrich the feature map and merge it as the output.

**Figure 7:**
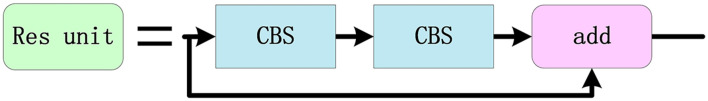
Res unit module.

**Figure 8:**
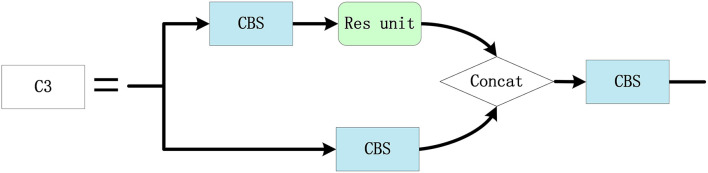
C3 module configuration.

The Res unit is a standard residual module, which is depicted in Figure 7. The 1 × 1 standard convolution is used to reduce the dimension, and the 3 × 3 convolution is used to extract features. Then, the original information and feature information after convolution are added as the output. The C3 module is used for feature extraction and feature fusion, as described in Figure 8. Hence, the rich semantic information and features are obtained to convolve the upper layer feature map based on the Res unit and the 3 × 3 convolution is applied to extract features. Then, 1 × 1 convolution is applied to reduce the dimension of the original feature map, which is spliced with the convolved feature maps and used as the output.

### 2.3. Feature fusion module

**Figure 9:**
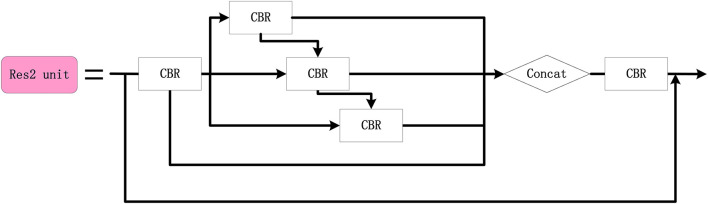
The configuration of the Res2 unit module.

**Figure 10:**
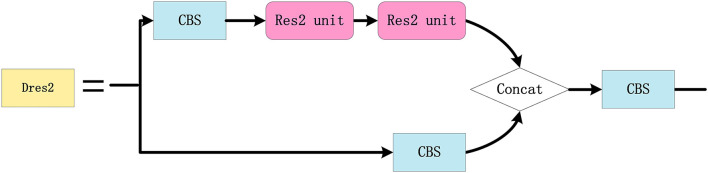
The configuration of the Dres2 module.

In order to improve the accuracy of the small target detection, the Res2 unit module is designed (refer to Figure 9), where multigroup 3 × 3 convolutions are cascaded to enlarge the receptive field of the network and the features of each group are fused. The Dres2 module is further designed based on the C3 module (refer to Figure 10), where the original residual block is replaced with two Res2 modules. Compared to the C3 module, the Dres2 module can increase the receptive field to obtain more multi-scale global information. Therefore, the Dres2 module is applied here to realize fine-grained feature extraction.

**Figure 11:**
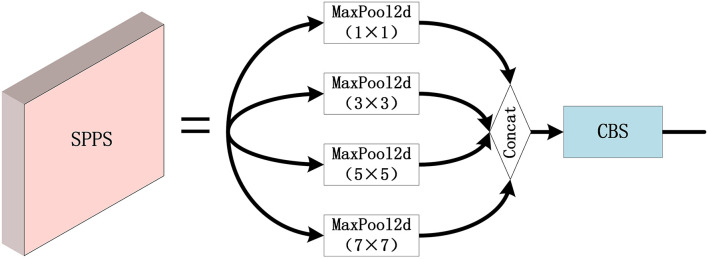
The configuration of the SPPS module.

As depicted in Figure 11, the SPPS module is a modified version of the Spatial Pyramid Pooling (SPP) module in the network, where the three groups of maximum pooling are 1 × 1, 3 × 3, 5 × 5, and 7 × 7. Since small targets have a relatively small proportion of pixels in the remote sensing images, the effective feature information may be difficult to extract. In order to overcome the above difficulty, the SPPS module applies different sizes of the max pooling kernels, and thus, the feature information of the small targets can be retained accordingly since SPPS not only has the advantages of SPP but also can improve the detection performance for small targets.

### 2.4. Input size of the network

The input image size of the YOLOv5 network is 640 × 640 and the output size is 80, 40, and 20 in the prediction head. Compared to the YOLOv5 algorithm, the input size of the network and the predicted feature map are maximized to 1,024 and 256, and 128 and 64, respectively. Consequently, the input size of the network is enlarged to overcome the limitation of less small target features in the remote sensing images.

### 2.5. Loss function

Here, the IoU and GIoU Loss functions of the original YOLOv5 algorithm are first presented to analyze the deficiencies in small target detection. Then, the EIoU Loss is introduced (Zhang et al., 2021), where the GIoU Loss function refers to an improved intersection-over-union (IoU). The IoU is used to denote the intersection ratio of the prediction box (*PB*) and ground truth box (*GB*), which is described as follows:


(1) $$\begin{array}{l} IoU=\frac{PB\cap GB}{PB\cup GB}, \end{array}$$

Moreover, the *IoU* Loss function is calculated as follows:


(2) $$\begin{array}{l} L_{IoU}=1-\frac{PB\cap GB}{PB\cup GB}. \end{array}$$

However, if there is no intersection between *PB* and *GB, IoU* Loss is nearly zero, which can hardly be used to reflect their distance. Moreover, the IoU Loss has a relatively slow convergence rate; hence, the *GIoU* is introduced to avoid such a problem, calculated as follows:


(3) $$\begin{array}{l} GIoU=IoU-\frac{A_{c}-U}{A_{c}}, \end{array}$$

where *A*_*c*_ is the area of the smallest rectangular box including both *PB* and *GB* simultaneously and *U* is the union of *PB* and *GB*. Furthermore, the GIoU Loss can be expressed as follows:


(4) $$\begin{array}{l} L_{GIoU}=1-GIoU=1-IoU+\frac{A_{c}-U}{A_{c}}. \end{array}$$

It is noted that *GIoU* Loss can be optimized for situations where the *PB* and *GB* are not overlapped. Nevertheless, if these two boxes are positioned relatively close, the values of the *GIoU* and *IoU* Loss are also approximately equal. In order to solve the above problem, the *EIoU* Loss is used as the loss function of LCB-YOLOv5. The *EIoU* and the *EIoU* loss functions are calculated as follows:


(5a) $$\begin{array}{lll} EIoU & = & IOU-\frac{\rho^{2}\left(b,b^{gt}\right)}{c^{2}}-\frac{\rho^{2}\left(w,w^{gt}\right)}{c_{w}^{2}}-\frac{\rho^{2}\left(h,h^{gt}\right)}{c_{h}^{2}}, \end{array}$$

(5b) $$\begin{array}{l} L_{EIoU}=L_{IoU}+L_{dis}+L_{asp}=1-IOU+\frac{\rho^{2}\left(b,b^{gt}\right)}{c^{2}}+\frac{\rho^{2}\left(w,w^{gt}\right)}{c_{w}^{2}}\\ \;+\frac{\rho^{2}\left(h,h^{gt}\right)}{c_{h}^{2}}, \end{array}$$

where *c*_*w*_ and *c*_*h*_ are the minimum widths and heights of the outer box covering two boxes, respectively. Compared with *IoU* and *GIoU* Loss functions, the distance between the target and anchor, the overlap rate and penalty items are considered based on the *EIoU* Loss function. Therefore, the regression accuracy for detection is more stable and the training convergence speed is faster.

## 3. Experimental results and analysis

### 3.1. Experimental settings

The proposed LCB-YOLOv5s network is trained with the RTX 3090, 24G memory, and Ubuntu 20.04.4 operating system, while the proposed network and the comparison algorithms are programmed in Python 3.8 and Cuda 11.3. The hyperparametric configuration is displayed in Table 1. In total, two datasets are selected for the experiments. The first is the VisDrone2019 dataset, which was collected by the Aiskyeyee team in the Machine Learning and Data Mining Laboratory of Tianjin University. It includes 10 categories comprising more than 2.6 million annotation boxes. The targets in the VisDrone2019 dataset are pedestrians, people, bicycles, cars, vans, trucks, tricycles, awning-tricycles, buses, and motors. Moreover, the training and validation sets contain 6,471 and 548 remote sensing images, respectively. The other dataset is the DIOR remote sensing dataset, which contains 20 categories with 23,463 remote sensing images and 192,472 examples.

**Table 1:** Hyperparametric configuration of the experiments.

**Hyperparametric**	**Epochs**	**Batch size**	**Learning rate**	**Momentum**	**Weight decay**
Configuration	150	16	0.01	0.973	0.0005

In the experiments, vehicles, ships, and airplanes are selected as the targets from 1,673 remote sensing images. Furthermore, a new dataset called the DIOR-VAS dataset is reconfigured including three types of targets: vehicles, airplanes, and ships. As shown in Table 2, the training and verification sets contain 1,334 and 339 remote sensing images, respectively.

**Table 2:** Details of the VisDrone2019 and DIOR datasets.

**Datasets**	**Categories**	**Totaling images**	**Training set**	**Validation set**
VisDrone2019	10	8,629	6,471	548
DIOR	20	23,463	5,862	5,863
DIOR-VAS	3	1,673	1,334	339

### 3.2. Evaluation metrics of the experiments

During the experiments, three common evaluation metrics are used to evaluate the effect of the proposed method, mean average precision (*mAP*), precision (*P*), and recall (*R*). Specifically, *P* and *R* are calculated as follows:


(6a) $$\begin{array}{l} P=\frac{TruePositives}{TruePositives+FalsePositives}, \end{array}$$

(6b) $$\begin{array}{l} R=\frac{TruePositives}{TruePositives+FalseNegatives}, \end{array}$$

where *TruePositives* denotes the targets correctly classified as positive examples, *FalsePositives* denotes the targets incorrectly classified as positive examples, and *FalseNegatives* denotes the targets incorrectly classified as negative examples.

Additionally, *AP* is the average classification accuracy of a category in the datasets. It is calculated as follows:


(7) $$\begin{array}{l} AP=\int_{0}^{1}P\left(R\right)dt \end{array}$$

where *P*(*R*) is the *P*–*R* curve to be used to calculate the *AP*. Based on the *AP*, the *mAP* can be obtained as follows:


(8) $$\begin{array}{l} mAP=\frac{\overset{N}{\underset{n=0}{\sum }}AP_{n}}{N} \end{array}$$

where *N* is the number of the detected target categories.

### 3.3. Experimental results and analysis

Table 3 displays the comparison results of our proposed method with the other five approaches, *Mets* = {YOLOv5, YOLOv3, YOLOv3-SPP, YOLOv7, PicoDet}, on the Visdrone2019 dataset. The proposed method has achieved significantly higher performance than the other methods, with *P, R*, and *mAP* as 56.2, 46.7, and 47.9, respectively. Particularly, the *mAP* of the proposed method is 17.4, 19.7, 22, 22.5, and 21.7 higher than those of the methods in *Mets* one by one. Furthermore, the *P* of the LCB-YOLOv5s is higher by {14, 20.5, 15.7, 13.7, 16.7} in comparison to those of methods in *Mets*. Moreover, the *R* of the LCB-YOLOv5s is higher by {15.2, 16.2, 19.9, 21.6, 16.4} than those of the methods in *Mets* in turn. However, the PicoDet method has a relatively weaker performance in the DIOR-VAS dataset. Furthermore, in Table 3, the LCB-YOLOv5s exhibits much better detection performance than the other five methods for bus and pedestrian detection and slightly better detection performance than the rest methods for plane and ship detection. In general, LCB-YOLOv5s can achieve higher small target detection performance with a reduced false detection rate.

**Table 3:** Comparison of the proposed method and other approaches based on the Visdrone2019 dataset.

**Models**	***P* (%)**	***R* (%)**	***mAP* (%)**	**Car**	**Bus**	**Pedestrian**
YOLOv5	42.2	31.5	30.5	0.72	0.38	0.39
PicoDet	35.7	30.5	28.2	0.75	0.33	0.38
YOLOv3	40.5	26.8	25.9	0.65	0.28	0.32
YOLOv3-SPP	42.5	25.1	25.4	0.65	0.26	0.32
YOLOv7	39.5	30.3	26.2	0.72	0.33	0.34
LCB-YOLOv5s	56.2	46.7	47.9	0.86	0.65	0.59

**Figure 12:**
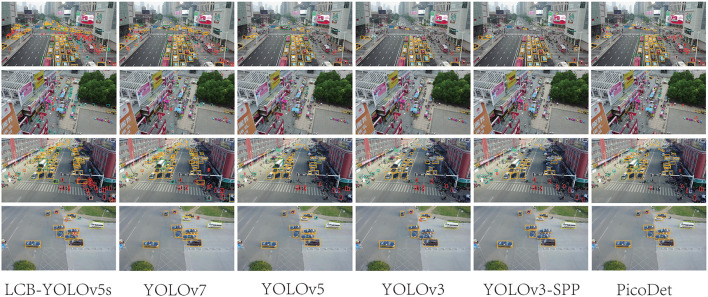
Comparison of the target detection of six different models on the Visdrone2019 dataset.

**Figure 13:**
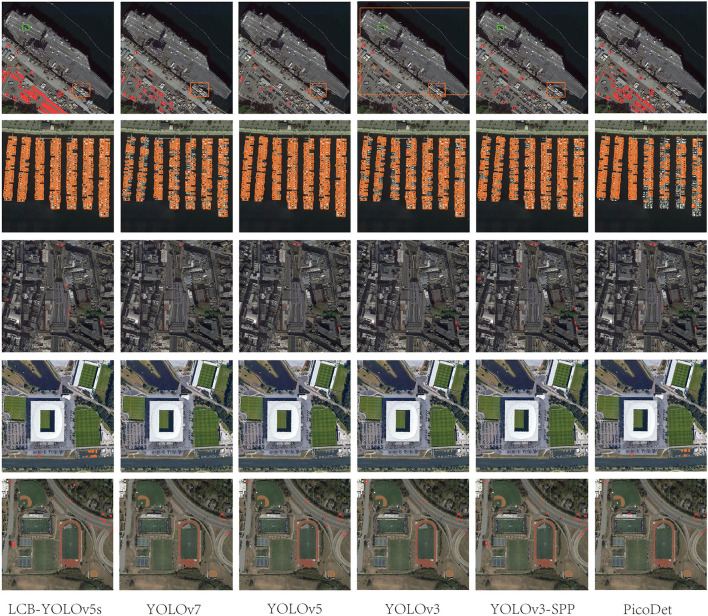
Comparison of the target detection of six different models on the DIOR-VAS dataset.

**Figure 14:**
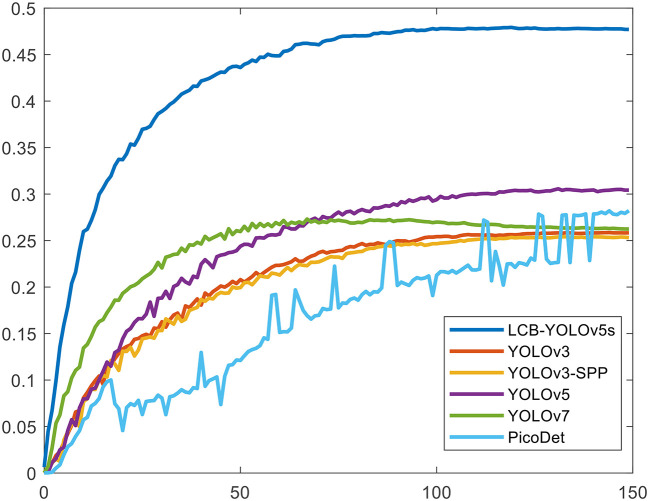
The *mAP* (threshold is 0.5) of the proposed method in comparison with the other five detection algorithms on the Visdrone2019 dataset.

**Figure 15:**
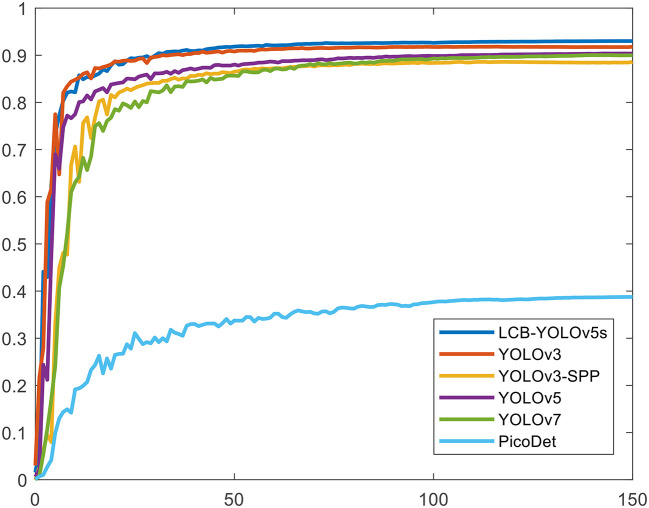
The *mAP* (the threshold is 0.5) of the proposed method in comparison with the other five detection algorithms on the DIOR-VAS dataset.

Table 4 illustrates the comparison results of the proposed method with the other five methods on the DIOR-VAS dataset, where vehicles, airplanes, and ships are selected as the small targets. The proposed method exhibits a better performance than the other methods, with *mAP, P*, and *R* of 93, 93.4, and 88.6, respectively. Particularly, the *mAP, P*, and *R* of YOLOv5s and YOLOv7 are 90.4, 93.3, and 85.8 and 90, 92.5, and 85.8, respectively. Thus, the *mPA* and *R* of the YOLOv3 and YOLOv3-SPP are lower by 4.4, 4.6, 4.4, and 4.7, respectively. The *R* of the YOLOv3 and YOLOv3-SPP is also relatively lower. Figure 12 displays the target detection results of the six algorithms on the Visdrone2019 dataset, where the orange, green, and red boxes indicate the detected targets of cars, buses, and pedestrians, respectively. Compared to the other five algorithms, LCB-YOLOv5s can accurately detect more targets, especially buses and pedestrians, although the prediction boxes are densely distributed in the leftmost subfigure of Figure 12. This demonstrates that the proposed LCB-YOLOv5 algorithm has an advantage over the other algorithms for small target detection. The target detection comparison of the six algorithms on the DIOR-VAS dataset is illustrated in Figure 13, where the orange, green, and red boxes are the detection results of the ships, airplanes, and vehicles. It is clear that more expected targets can be detected *via* LCB-YOLOv5s compared to the other methods. Additionally, Figures 14, 15 display the *mAP* (threshold is 0.5) of the six algorithms on the Visdrone2019 and DIOR-VAS datasets. The visual results of the original YOLOv5s and the LCB-YOLOv5s are demonstrated in Table 5. It can be intuitively seen that the proposed LCB-YOLOv5 algorithm has a better performance and higher robustness for small target detection in remote sensing images. In particular, the LCB-YOLOv5s have a stronger ability in dense small target detection.

**Table 4:** Comparison of the proposed method with other approaches based on the DIOR-VAS dataset.

**Models**	***P* (%)**	***R* (%)**	***mAP* (%)**	**Vehicle**	**Airplane**	**Ship**
YOLOv5	93.3	85.8	90.4	0.75	0.99	0.96
PicoDet	81.6	29.3	55.9	0.53	0.54	0.59
YOLOv3	92.7	84	88.6	0.74	0.99	0.93
YOLOv3-SPP	92.9	83.9	88.6	0.74	0.98	0.94
YOLOv7	92.5	85.8	90	0.74	0.99	0.96
LCB-YOLOv5s	93.4	88.6	93	0.84	0.99	0.96

**Table 5:** Visual results of the small target detection on Visdrone2019 dataset.

**Categories**	**Visual results of YOLOv5s**	**Visual results of LCB-YOLOv5s**
The original images	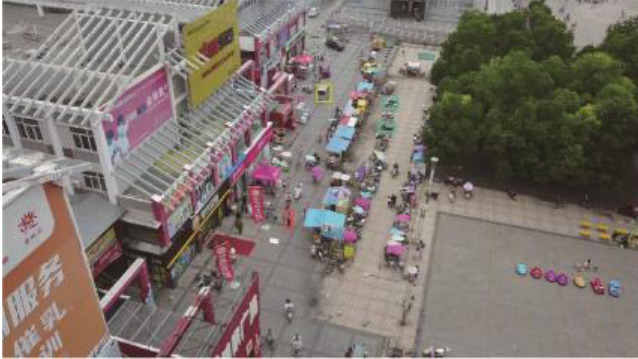	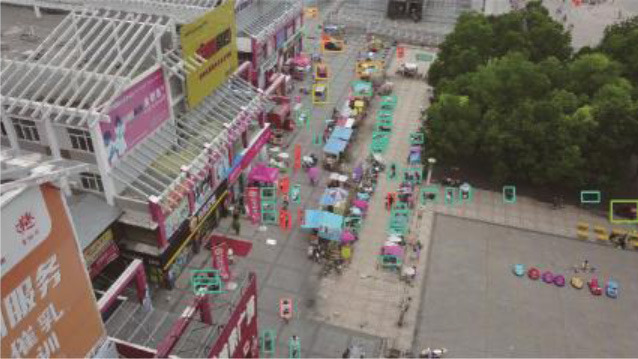
Backbone	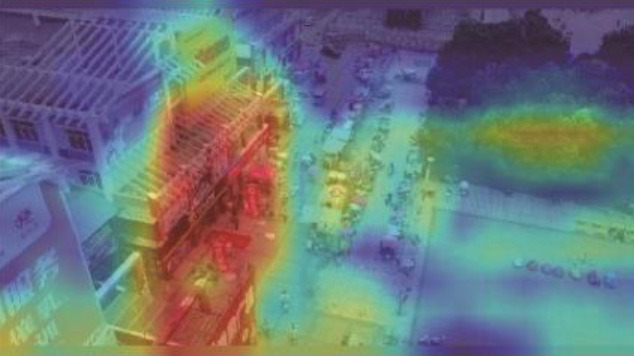	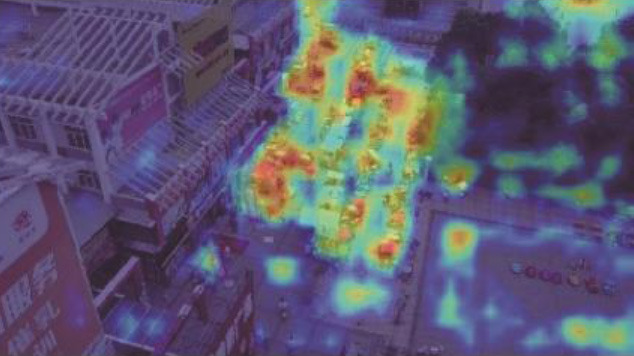
Prediction head 1	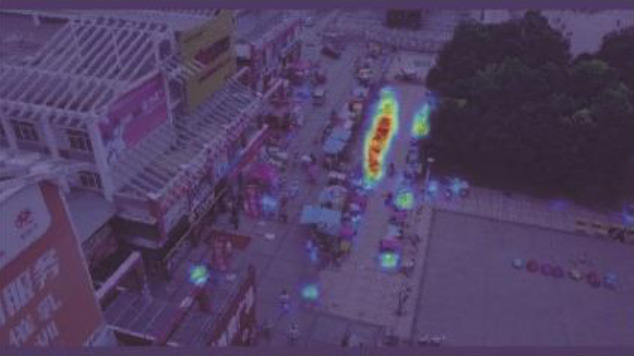	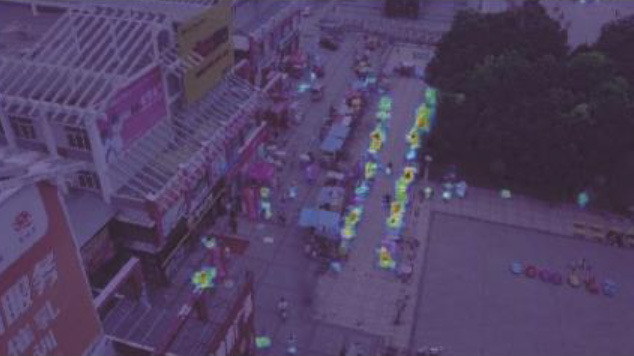
Prediction head 2	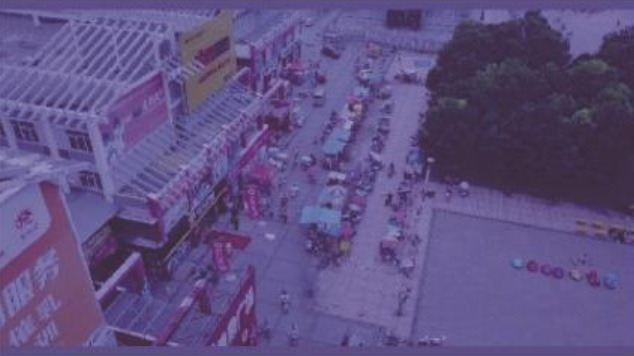	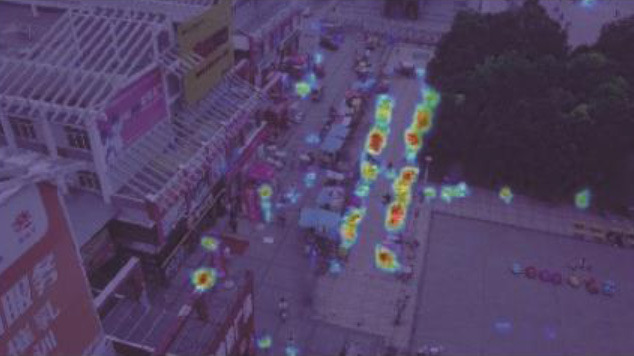
Prediction head 3	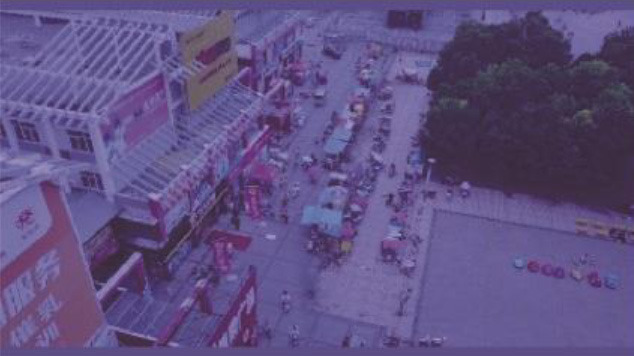	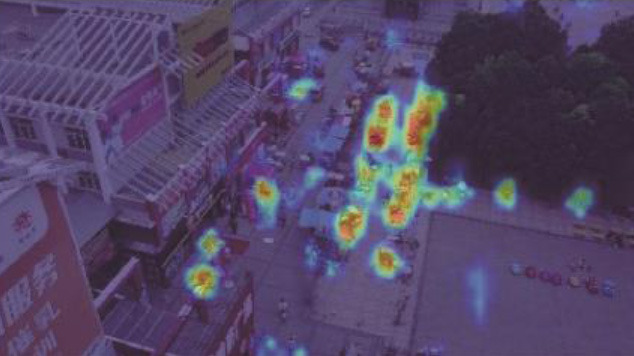

### 3.4. Results of ablation experiments

Ablation experiments are further performed to verify the optimization performance of each improved module. The EIoU loss function, LCB module, SPPS module, and Dres2 Module are introduced in the original network to construct the improved Model 1, improved Model 2, improved Model 3, and improved Model 4, respectively. In the improved model 5, the input size is 1,024, while all the mentioned modifications above are applied in the improved Model 6. The ablation results with the improved modules are listed in Table 6.

**Table 6:** Results of ablation experiments.

**Model**	**EIOU**	**LCB**	**SPPS**	**Dres2**	**Input 1,024**	***mAP***	**Improvement** **(*mAP*)**
LCB-YOLOv5s	×	×	×	×	×	30.5	-
Improved Model 1 (IM1)	√	×	×	×	×	31.8	+1.3
Improved Model 2 (IM2)	×	√	×	×	×	42.3	+11.8
Improved Model 3 (IM3)	×	×	√	×	×	31.4	+0.9
Improved Model 4 (IM4)	×	×	×	√	×	31.1	+0.6
Improved Model 5 (IM5)	×	×	×	×	√	43.3	+12.8
Improved Model 6 (IM6)	√	√	√	√	√	47.9	+17.4

Compared with the original YOLOv5s network, the *mAP* of the model is improved by 1.3 percentage points in IM1, and the *mAP* of the models with IM3 and IM4 is increased by 0.9 and 0.6 percentage points, respectively. Moreover, the *mAP* of the model is improved by 11.8 percentage points with IM2. Meanwhile, when the input size is 1,024, the *mAP* of IM5 is also improved by 12.8 percentage points. Furthermore, when these six improvements are combined in IM6, the *mAP* is increased by 17.4 percentage points. The ablation experimental results strongly demonstrate that the proposed LCB-YOLOv5s model has a higher detection performance for small target detection with remote sensing images.

## 4. Conclusion

In this paper, an improved detection algorithm, called LCB-YOLOv5s, has been developed to detect small target objects in remote sensing images. The proposed algorithm comprises the LCB module *via* the combination of LSM and C3 modules, the SPPS module, and the Dres2 module in the feature extraction module to achieve multi-scale feature fusion. Furthermore, the input size of the network is increased and the output feature map size is set in various scales to improve the small target detection performance. Experiments have been performed on the DIOR and Visdrone2019 datasets to compare with other methods to verify the effectiveness of the proposed method for small target detection. Future work will continue to investigate small target detection and tracking under special and harsh circumstances with more general remote sensing datasets.

## Data availability statement

The original contributions presented in the study are included in the article/supplementary material, further inquiries can be directed to the corresponding author.

## Author contributions

Conceptualization and revising: WP, ZS, and KG. Methodology, experiments, and writing the original: WP and ZS. All authors have read and agreed to the published version of the manuscript.

## References

[B1] Chen S.-B. Wei Q.-S. Wang W.-Z. Tang J. Luo B. Wang Z.-Y.. (2021). Remote sensing scene classification *via* multi-branch local attention network. IEEE Trans. Image Process. 31, 99–109. 10.1109/TIP.2021.3127851 34793302

[B2] Dong X. Tian J. Tian Q. (2022). A feature fusion airport detection method based on the whole scene multispectral remote sensing images. IEEE J. Sel. Top. Appl. Earth Observ. Remote Sens. 15, 1174–1187. 10.1109/JSTARS.2021.3139926

[B3] Du B. Sun Y. Cai S. Wu C. Du Q. (2018). Object tracking in satellite videos by fusing the kernel correlation filter and the three-frame-difference algorithm. IEEE Geosci. Remote Sens. Lett. 15, 168–172. 10.1109/LGRS.2017.2776899

[B4] Fan J. Lee J. H. Jung I. S. Lee Y. K. (2021). “Improvement of object detection based on Faster R-CNN and YOLO,” in 2021 36th International Technical Conference on Circuits/Systems, Computers and Communications (Jeju), 1–4.

[B5] Fu J. Sun X. Wang Z. Fu K. (2020). An anchor-free method based on feature balancing and refinement network for multiscale ship detection in SAR images. IEEE Trans. Geosci. Remote Sens. 59, 1331–1344. 10.1109/TGRS.2020.3005151

[B6] Guo Y. Jia X. Paull D. (2017). “A domain-transfer support vector machine for multi-temporal remote sensing imagery classification,” in 2017 IEEE International Geoscience and Remote Sensing Symposium (Fort Worth, TX: IEEE), 2215–2218.

[B7] Habibzadeh M. Ameri M. Sadat Haghighi S. M. Ziari H. (2022). Application of artificial neural network approaches for predicting accident severity on rural roads (case study: tehran-qom and tehran-saveh rural roads). Math. Probl. Eng. 2022, 521470. 10.1155/2022/5214703

[B8] Han X. Zhong Y. Zhang L. (2017). An efficient and robust integrated geospatial object detection framework for high spatial resolution remote sensing imagery. Remote Sens. 9, 666. 10.3390/rs9070666

[B9] He K. Zhang X. Ren S. Sun J. (2016). “Deep residual learning for image recognition,” in Proceeding of IEEE Conference on Computer Vision and Pattern Recognition (IEEE), 770–778.

[B10] Hou B. Ren Z. Zhao W. Wu Q. Jiao L. (2020). Object detection in high-resolution panchromatic images using deep models and spatial template matching. IEEE Trans. Geosci. Remote Sens. 58, 956–970. 10.1109/TGRS.2019.2942103

[B11] Hu Y. Li X. Zhou N. Yang L. Peng L. Xiao S.. (2019). A sample update-based convolutional neural network framework for object detection in large-area remote sensing images, IEEE Geosci. Remote Sens. Lett. 16, 947–951. 10.1109/LGRS.2018.2889247

[B12] Huang L. Chen C. Yun J. Sun Y. Tian J. Hao Z.. (2022). Multi-scale feature fusion convolutional neural network for indoor small target detection. Front. Neurorobot. 16, 881021. 10.3389/fnbot.2022.881021 35663726 PMC9160233

[B13] Huang Z. Li W. Xia X. Wang H. Jie F. Tao R.. (2022). LO-Det: lightweight oriented object detection in remote sensing images. IEEE Trans. Geosci. Remote Sens. 60, 5603515. 10.1109/TGRS.2021.3067470

[B14] Jia Y. (2000). Robust control with decoupling performance for steering and traction of 4WS vehicles under velocity-varying motion. IEEE Trans. Control Syst. Technol. 8, 554–569. 10.1109/87.845885

[B15] Jia Y. (2003). Alternative proofs for improved lmi representations for the analysis and the design of continuous-time systems with polytopic type uncertainty: a predictive approach. IEEE Trans. Autom. Control. 48, 1413–1416. 10.1109/TAC.2003.815033

[B16] Jiang W. Zhao L. Wang Y. Liu W. Liu B.-D. (2021). U-Shaped attention connection network for remote-sensing image super-resolution. IEEE Geosci. Remote Sens. Lett. 19, 1–5. 10.1109/LGRS.2021.3127988

[B17] Lawal M. O. (2021). Tomato detection based on modified YOLOv3 framework. Sci. Rep. 11, 1–11. 10.1038/s41598-021-81216-5 33446897 PMC7809275

[B18] Li H. Man Y. (2016). “Moving ship detection based on visual saliency for video satellite,” in 2016 IEEE International Geoscience and Remote Sensing Symposium (Beijing: IEEE), 1248–1250.

[B19] Li K. Wan G. Cheng G. Meng L. Han J. (2020). Object detection in optical remote sensing images: a survey and a new benchmark. ISPRS J. Photogramm. Remote Sens. 159, 296–307. 10.1016/j.isprsjprs.2019.11.023

[B20] Li N. Cheng L. Huang L. Ji C. Jing M. Duan Z.. (2021). Framework for unknown airport detection in broad areas supported by deep learning and geographic analysis. IEEE J. Sel. Top. Appl. Earth Observ. Remote Sens. 14, 6328–6338. 10.1109/JSTARS.2021.3088911

[B21] Li S. Xu Y. Zhu M. Ma S. Tang H. (2019). Remote sensing airport detection based on end-to-end deep transferable convolutional neural networks. IEEE Geosci. Remote Sens. Lett. 16, 1640–1644. 10.1109/LGRS.2019.2904076

[B22] Ling J. Wang H. Xu M. Chen H. Li H. Peng J.. (2022). Mathematical study of neural feedback roles in small target motion detection. Front. Neurorobot. 16, 984430. 10.3389/fnbot.2022.984430 36203523 PMC9530796

[B23] Liu Y. Liao Y. Lin C. Jia Y. Li Z. Yang X.. (2022). Object tracking in satellite videos based on correlation filter with multi-feature fusion and motion trajectory compensation. Remote Sens. 14, 2022. 10.3390/rs14030777

[B24] Lu X. Ji J. Xing Z. Miao X. (2021). Attention and feature fusion SSD for remote sensing object detection. IEEE Trans. Instrum. Meas. 70, 1–9. 10.1109/TIM.2021.311809233776080

[B25] Lu X. Zhang Y. Yuan Y. Feng Y. (2020). Gated and axis-concentrated localization network for remote sensing object detection. IEEE Trans. Geosci. Remote Sens. 58, 179–192. 10.1109/TGRS.2019.2935177

[B26] Ma H. Wang Y. (2022). Full information H2 control of borel-measurable Markov jump systems with multiplicative noises. Mathematics 10, 37. 10.3390/math10010037

[B27] Meng L. and Kerekes, J. P.. (2012). Object tracking using high resolution satellite imagery. IEEE J. Sel. Top. Appl. Earth Observ. Remote Sens. 5, 146–152. 10.1109/JSTARS.2011.2179639

[B28] Mikriukov G. Ravanbakhsh M. Demir B. (2022). “Deep unsupervised contrastive hashing for large-scale cross-modal text-image retrieval in remote sensing,” in 2022 IEEE International Conference on Acoustics, Speech and Signal Processing (Singapore: IEEE), 4463–4467.

[B29] Pei W. (2022). Staring imaging attitude tracking control laws for video satellites based on image information by hyperbolic tangent fuzzy sliding mode control. Comput. Intell. Neurosci. 2022, 8289934. 10.1155/2022/8289934 36110911 PMC9470366

[B30] Pei W. Lu X. (2022). Moving object tracking in satellite videos by kernelized correlation filter based on color-name features and Kalman prediction. Wirel. Commun. Mob. Comput. 2022, 9735887. 10.1155/2022/9735887

[B31] Shakarami A. Menhaj M. B. Mahdavi-Hormat A. Tarrah H. (2021). A fast and yet efficient YOLOv3 for blood cell detection. Biomed. Signal Process. Control 66, 102495. 10.1016/j.bspc.2021.102495

[B32] Shi P. Jiang Q. Shi C. Xi J. Tao G. Zhang S.. (2021). Oil well detection *via* large-scale and high-resolution remote sensing images based on improved YOLO v4. Remote Sens. 13, 3243. 10.3390/rs13163243

[B33] Tu J. Gao F. Sun J. Hussain A. Zhou H. (2021). Airport detection in sar images *via* salient line segment detector and edge-oriented region growing. IEEE J. Sel. Top. Appl. Earth Observ. Remote Sens. 14, 314–326. 10.1109/JSTARS.2020.3036052

[B34] Wang J. Wang Y. Wu Y. Zhang W. Wang Q. (2020). FRPNet: a feature-reflowing pyramid network for object detection of remote sensing images. IEEE Geosci. Remote Sens. Lett. 99, 1–5. 10.1109/LGRS.2020.3040308

[B35] Wang M. Dong Z. Cheng Y. Li D. (2018). Optimal segmentation of high-resolution remote sensing image by combining superpixels with the minimum spanning tree. IEEE Trans. Geosci. Remote Sens. 56, 228–238. 10.1109/TGRS.2017.2745507

[B36] Wang P. Sun X. Diao W. Fu K. (2020). Fmssd: feature-merged single-shot detection for multiscale objects in large-scale remote sensing imagery. IEEE Trans. Geosci. Remote Sens. 58, 3377–3390. 10.1109/TGRS.2019.2954328

[B37] Wu W. Yin Y. Wang X. Xu D. (2018). Face detection with different scales based on faster R-CNN. IEEE T. Cybern. 49, 4017–4028. 10.1109/TCYB.2018.2859482 30113907

[B38] Xu D. and Wu, Y.. (2020). Improved YOLO-V3 with DenseNet for multi-scale remote sensing target detection. Sensors 20, 4276. 10.3390/s20154276 32751868 PMC7435986

[B39] Yin S. Li H. Teng L. (2020). Airport detection based on improved Faster RCNN in large scale remote sensing images. Sens. Imaging 21, 1–13. 10.1007/s11220-020-00314-2

[B40] Yu Y. Yang X. Li J. Gao X. (2022). A cascade rotated anchor-aided detector for ship detection in remote sensing images. IEEE Trans. Geosci. Remote Sens. 60, 5600514. 10.1109/TGRS.2020.3040273

[B41] Zhang F. Du B. Zhang L. Xu M. (2016). Weakly supervised learning based on coupled convolutional neural networks for aircraft detection. IEEE Trans. Geosci. Remote Sens. 54, 5553–5563. 10.1109/TGRS.2016.2569141

[B42] Zhang G. Lu S. Zhang W. (2019). Cad-net: a context-aware detection network for objects in remote sensing imagery. IEEE Trans. Geosci. Remote Sens. 57, 10015–10024. 10.1109/TGRS.2019.2930982

[B43] Zhang P. Niu X. Dou Y. Xia F. (2017). Airport detection on optical satellite images using deep convolutional neural networks. IEEE Geosci. Remote Sens. Lett. 14, 1183–1187. 10.1109/LGRS.2017.2673118

[B44] Zhang Y. Ren W. Zhang Z. Jia Z. Wang L. Tan T.. (2021). Focal and efficient IOU loss for accurate bounding box regression. Neurocomputing 506, 146–157. 10.1016/j.neucom.2022.07.042

[B45] Zhong Y. Zheng Z. Ma A. Lu X. Zhang L. (2020). Color: cycling, offline learning, and online representation framework for airport and airplane detection using gf-2 satellite images. IEEE Trans. Geosci. Remote Sens. 58, 8438–8449. 10.1109/TGRS.2020.2987907

[B46] Zhou L. Deng G. Li W. Mi J. Lei B. (2021). A lightweight SE-YOLOv3 network for multi-scale object detection in remote sensing imagery. Int. J. Pattern Recognit. Artif. Intell. 35, 2150037. 10.1142/S0218001421500373

[B47] Zhu P. Du D. Wen L. Bian X. Ling H. Hu Q.. (2019). “Visdrone-vid2019: The vision meets drone object detection in video challenge results,” in Proceedings of the IEEE/CVF International Conference on Computer Vision Workshops (Seoul: IEEE), 227–235.

[B48] Zhu X. Tuia D. Mou L. Xia G.-S. Zhang L. Xu F.. (2017). Deep learning in remote sensing: a comprehensive review and list of resources. IEEE Geosci. Remote Sens. Mag. 5, 8–36. 10.1109/MGRS.2017.2762307

