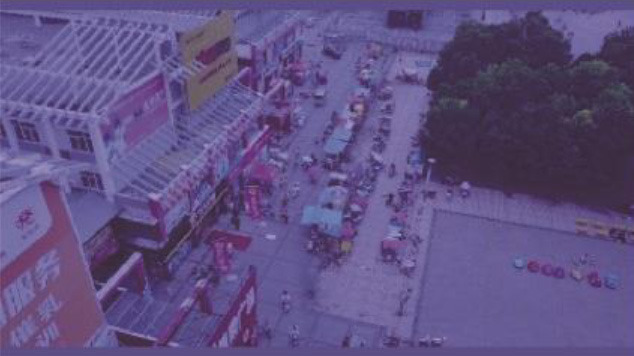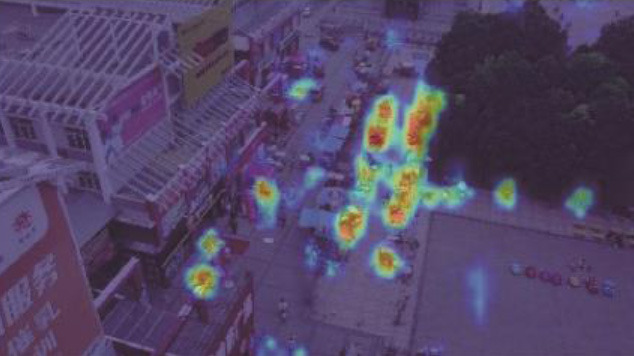# Corrigendum: Small target detection with remote sensing images based on an improved YOLOv5 algorithm

**DOI:** 10.3389/fnbot.2023.1161652

**Published:** 2023-02-28

**Authors:** Wenjing Pei, Zhanhao Shi, Kai Gong

**Affiliations:** ^1^The Seventh Research Division and the Center for Information and Control, School of Automation Science and Electrical Engineering, Beihang University (BUAA), Beijing, China; ^2^School of Information Science and Engineering, Shandong Agriculture and Engineering University, Jinan, China

**Keywords:** small target detection, remote sensing images, YOLOv5s, deep learning, EIoU loss

In the published article, there was an error in Figure 12, Figure 13, and Table 5 as published. The descriptions at the bottom of the Figure 12 and Figure 13 were deleted. In addition, the resolution of all the figures in Table 5 was not high enough. The corrected Figure 12, Figure 13, and Table 5 and their captions appear below.

The authors apologize for this error and state that this does not change the scientific conclusions of the article in any way. The original article has been updated.

**Figure 12:**
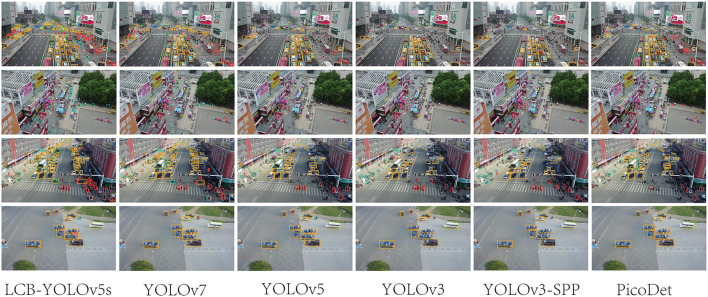
Comparison of the target detection of six different models on the Visdrone2019 dataset.

**Figure 13:**
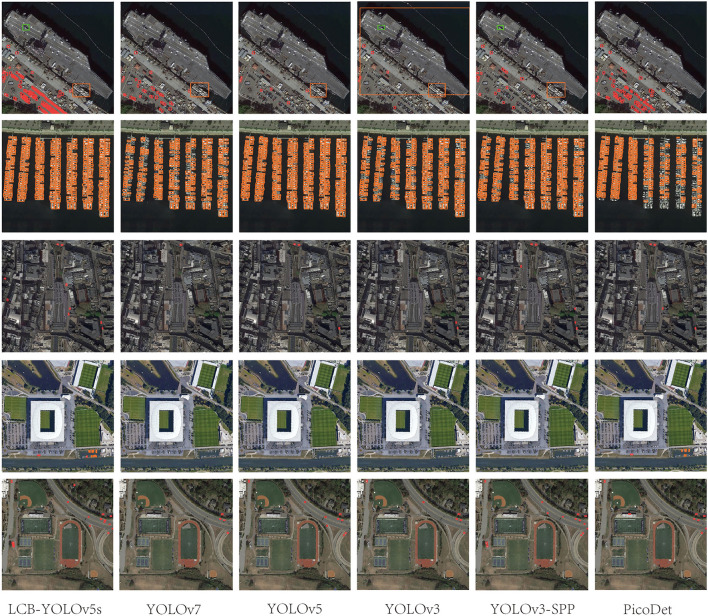
Comparison of the target detection of six different models on the DIOR-VAS dataset.

**Table 5:** Visual results of the small target detection on Visdrone2019 dataset.

**Categories**	**Visual results of YOLOv5s**	**Visual results of LCB-YOLOv5s**
The original images	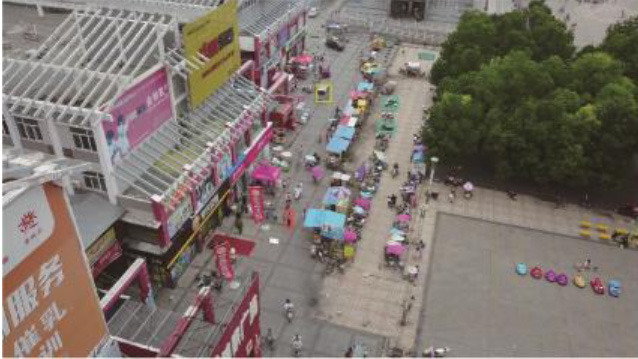	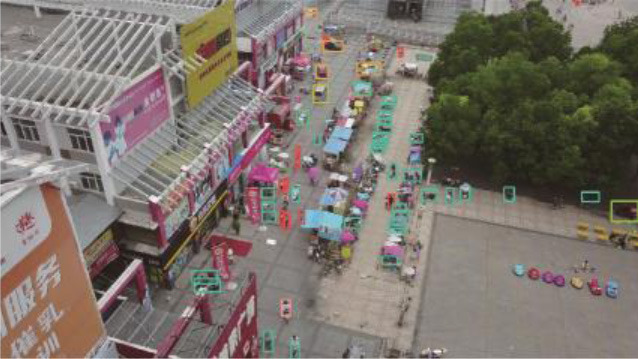
Backbone	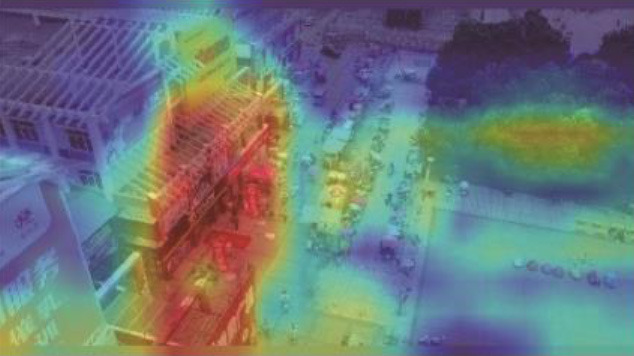	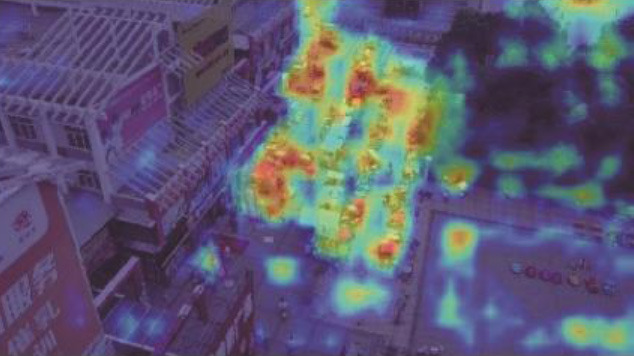
Prediction head 1	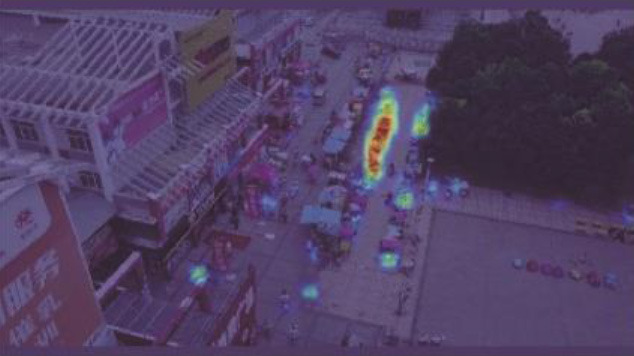	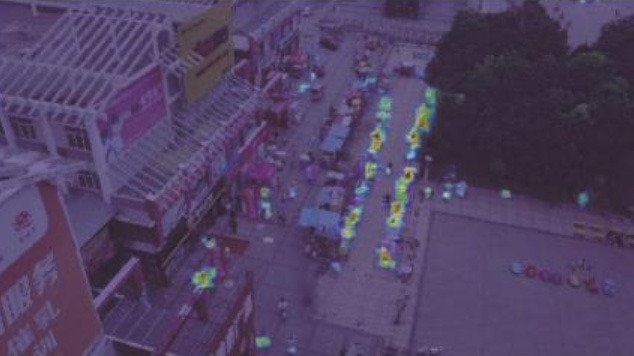
Prediction head 2	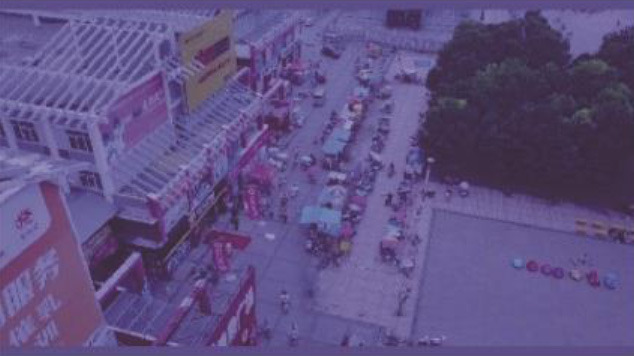	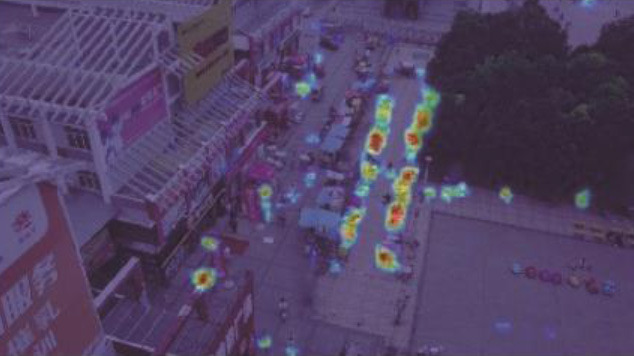
Prediction head 3